# Changes in gut microbiota composition after 12 weeks of a home-based lifestyle intervention in breast cancer survivors during the COVID-19 lockdown

**DOI:** 10.3389/fonc.2023.1225645

**Published:** 2023-09-01

**Authors:** Sabrina Donati Zeppa, Valentina Natalucci, Deborah Agostini, Luciana Vallorani, Stefano Amatori, Davide Sisti, Marco B. L. Rocchi, Valerio Pazienza, Francesco Perri, Annacandida Villani, Elena Binda, Concetta Panebianco, Gandino Mencarelli, Luigi Ciuffreda, Carlo Ferri Marini, Giosué Annibalini, Francesco Lucertini, Alessia Bartolacci, Marta Imperio, Edy Virgili, Vincenzo Catalano, Giovanni Piccoli, Vilberto Stocchi, Rita Emili, Elena Barbieri

**Affiliations:** ^1^ Department of Biomolecular Sciences, University of Urbino Carlo Bo, Urbino, Italy; ^2^ Division of Gastroenterology, Fondazione IRCCS “Casa Sollievo della Sofferenza” Hospital, San Giovanni Rotondo, Italy; ^3^ Cancer Stem Cells Unit, Institute for Stem Cell Biology, Regenerative Medicine and Innovative Therapeutics (ISBReMIT), IRCSS Casa Sollievo della Sofferenza, Opera di San Pio da Pietrelcina, San Giovanni Rotondo, Italy; ^4^ Breast Surgery Unit, Fondazione IRCCS Casa Sollievo della Sofferenza, San Giovanni Rotondo, Italy; ^5^ School of Biosciences and Veterinary Medicine, University of Camerino, Camerino, Italy; ^6^ U.O.C. Oncologia Medica, ASUR Area Vasta 1, Ospedale Santa Maria della Misericordia di Urbino, Urbino, Italy; ^7^ Università Telematica San Raffaele, Rome, Italy

**Keywords:** breast cancer survivors, COVID-19, gut microbiota, Mediterranean diet, physical exercise

## Abstract

**Background:**

Breast cancer (BC) is the second-leading cause of cancer-related death worldwide. This study aimed to investigate the effects of a 12-week home-based lifestyle intervention (based on nutrition and exercise) on gut microbial composition in twenty BC survivors of the MoviS clinical trial (protocol: NCT 04818359).

**Methods:**

Gut microbiota analysis through 16S rRNA gene sequencing, anthropometrics, Mediterranean Diet (MD) adherence, and cardiometabolic parameters were evaluated before (Pre) and after (Post) the lifestyle intervention (LI).

**Results:**

Beneficial effects of the LI were observed on MD adherence, and cardiometabolic parameters (pre vs post). A robust reduction of Proteobacteria was observed after LI, which is able to reshape the gut microbiota by modulating microorganisms capable of decreasing inflammation and others involved in improving the lipid and glycemic assets of the host. A significant negative correlation between fasting glucose and *Clostridia_vadinBB60* (r = -0.62), insulin and homeostatic model assessment (HOMA) index and *Butyricicoccus* genera (r = -0.72 and -0.66, respectively), and HDL cholesterol and *Escherichia/Shigella* (r = -0.59) have been reported. Moreover, positive correlations were found between MD adherence and *Lachnospiraceae_ND3007* (r = 0.50), *Faecalibacterium* (r = 0.38) and *Butyricimonas* (r = 0.39).

**Conclusion:**

These data suggest that adopting a healthy lifestyle, may contribute to ameliorate several biological parameters that could be involved in the prevention of cancer relapses through the modulation of gut microbiota.

## 1 Introduction

Breast cancer (BC) is the most common malignancy in women worldwide and continues to be a cause of death despite screening, early diagnosis, and personalized treatments. Incidence rates of BC have been increasing by about 0.5% per year, and the expected worldwide incidence of BC by 2050 is estimated to be 3.2 million new cases per year (1, 2). In Italy, there are around 55000 newly diagnosed cases of cancer each year, with 13000 resulting in death. These cases account for 14.6% of new cancer diagnoses (3, 4). From an etiological point of view, the onset of BC can be influenced by genetics or epigenetics (e.g., Micro-RNA miR-21, which controls various genes involved in tumor progression) and age (young age is a negative risk factor for BC). It can lead to advanced stages or lower survival rates, reproductive factors (the breast cells differentiation process during pregnancy could be protective), and lifestyle (e.g., less physical activity) (1, 2). This worrying data reflects the need for rigorous prevention and treatment strategies and further studies to understand all possible factors influencing the development of BC and its recurrence.

The gut microbiota is a newly emerging field of study as an additional environmental risk factor in a patient with BC, especially associated with metabolic dysfunctions. Recent evidence has described the role of gut microbiota dysbiosis in the development of BC via estrogen-dependent mechanisms linking the production of microbial-derived metabolites, adaptive immune response, and estrogen metabolism (5). Moreover, microbial metabolites have been demonstrated to express anticancer properties, including lithocholic acid, butyrate, and cadaverine (6, 7). Novel approaches targeting the gut microbiota have been proposed to preserve or restore normobiosis in the prevention and treatment of BC (7). The gut microbiota is influenced by several factors: some are unmodifiable (e.g., age, genetics, sex), and others are modifiable (e.g., lack of a regular diet and physical activity) (8). In particular, diet is a key component of the relationship between humans and their microbial residents and plays an important role in modulating its composition and diversity, affecting human health through this interaction (9). Changes in the host’s eating habits are reflected in the bacterial metabolism and induce the proliferation of the species most suitable for the use of the nutrients consumed (10). A western-type diet, rich in fat, salt, meat, refined flour, and sugar, can induce a state of dysbiosis, which is a change in the structural and functional balance of the gut microbial population often associated with obesity, diabetes and chronic inflammation (11). It has been recognized that 35% of all cancers are associated with dietary intake, including 50% of breast carcinomas (12), and it is likely that microbiota can be involved in carcinogenesis. On the other hand, correct dietary habits can positively influence intestinal microbiota, restore gut eubiosis, and hence improve overall host health.

The Mediterranean Diet (MD) is recognized by UNESCO as a cultural heritage of humanity. It is considered one of the healthiest nutritional patterns because it prefers raw foods to processed ones, and it provides a high consumption of vegetables, fruits, whole grains, unsaturated fats such as olive oil, fish, eggs, nuts, legumes, and poultry, reducing red meat and sugars (13). Along with protection against diabetes and metabolic diseases, a reduction in BC has also been shown (14). It has been demonstrated that MD improves the microbiota composition compared to other diets (15). Moreover, Pellegrini et al. (16) demonstrated that MD, in addition to probiotics, can positively affect gut microbiota composition and that these effects translate into the improvement of metabolic and anthropometric parameters.

Low saturated and high ω-3 fatty acids intake has been linked to anti-inflammatory signaling, which is also mediated by microbiota (17, 18). High amounts of fibers and polyphenols that exert a prebiotic action on specific strains lead to increased short-chain fatty acids (SCFA), lowering cancer risk and cardio-metabolic diseases (19, 20). Fermentable fibers allow bacteria to produce butyrate, acetate, and propionate (21) that regulate immunity and metabolism via microbiota (22). The protection from cancer seems to be improved by increased *Bifidobacterium* genera (23) and decreased *Fusobacterium nucleatum* (15). Furthermore, adherence to the MD increases α-diversity, which expresses the number of species present in the microbiota and positively correlates with overall health (24).

In both animal and human investigations, physical exercise was associated with favorable modifications in the gut microbiota’s diversity, richness, and composition (25). Positive associations have been shown in humans between butyrate-producing bacteria, α-diversity, and cardiorespiratory fitness (26), as well as between athletes and sedentary controls in terms of SCFA concentrations and higher turnover of carbohydrates and proteins. When fecal samples from nineteen people belonging to three cohorts (adults elite, youth elite, and youth non-elite athletes) were analyzed, it was found that top athletes had higher microbial diversity, a distinct taxonomic and functional composition, and a substantial correlation with athletic performance (27). Exercise alone does not account for changes in the microbiota composition; these changes also have to do with nutritional consumption, which research has shown to be altered by exercise itself (28). In a study by Donati Zeppa et al. (29), nine weeks of high-intensity exercise caused a shift in the gut microbial population towards a healthier microbiome in healthy male college students. Moreover, changes in the gut microbiota composition were correlated with dietary, body composition, and performance variables. Cheng et al. (30) recently demonstrated that a combined 8.6-month aerobic exercise and low-carbohydrate dietary intervention increased microbiome diversity and stability in patients with nonalcoholic fatty liver disease. Further, Furber et al. (31) reported a significant association between microbial stability following a dietary intervention and athletic performance in a cohort of highly trained athletes.

Several studies have been carried out on the role of microbiota in human health, and particular attention should be paid to the frail population. In this regard, BC survivors represent a population in which the combined influence of cancer treatment and advancing age often coincides with several physiological changes harmful to cardio-metabolic health, including increased adiposity (32), dysbiosis (33), and reduced cardiorespiratory fitness (34). When BC survivors complete primary treatments (i.e., chemotherapy and radiotherapy), women face several challenges in the long term, and some of them continue with hormone therapy for 5 to 10 years. Side effects of treatments include significant changes in anxiety, fatigue, and sleep dysfunction (35), as well as changes in the metabolic profile (36). Thus, interventions based on diet and physical activity have the potential to reduce comorbidity and prevent BC recurrences (37, 38). An increasing need for such lifestyle interventions was highlighted during the pandemic emergency, which further increased the health risks for people with a poor lifestyle (39, 40).

We previously reported that a 12-week home-based lifestyle intervention focused on MD and an aerobic exercise training program performed during the COVID-19 home confinement significantly ameliorated cardiometabolic parameters in BC survivors (41). Here we report the gut microbial composition before and after the lifestyle intervention in twenty BC survivors. The present study explores the effect of lifestyle (MD and aerobic exercise) in modulating the gut microbial composition in twenty BC survivors during the first wave of the COVID-19 lockdown.

## 2 Methods

### 2.1 Study design and population

The MoviS clinical trial (protocol no. NCT04818359) was designed as an open-label randomized controlled trial with two parallel groups (1:1 randomization ratio with the control arm). However, due to the imposed COVID-19 pandemic restrictions, after the approval of the institutional ethics committee, the study protocol was amended (protocol no. 29/20, 22/04/2020). As reported by Natalucci et al. (41), the forced changes in the study protocol made the difference in cardiometabolic parameters between the intervention and control arms negligible, providing similar adaptations between groups. Therefore, due to the lack of meaningful differences between the two interventions, the results of the two groups were combined. Informed consent was obtained from all individual participants included in the study, and the research was performed in accordance with the Declaration of Helsinki. The women included in this study were recruited at the “*Santa Maria della Misericordia*” Hospital of Urbino (Italy), as they were eligible for the inclusion and exclusion criteria reported in https://clinicaltrials.gov/ct2/show/NCT04818359. A sample size was considered (n=20), according to a previously published paper (35).

### 2.2 Lifestyle intervention

Following surgery and primary therapies (chemotherapy and/or radiotherapy), eligible participants participated in a lifestyle intervention. As previously described, participants (intervention and control arms) received 12-week lifestyle (nutrition and exercise) educational counseling and program (41). All participants received nutritional advice based on MD during the intervention phase, while only the intervention arm participated in a 12-week exercise training program (2 on-site and 1 remotely supervised aerobic session per week) with progressive increases in exercise intensity (from 40% to 70% of heart rate reserve) and duration (from 20 to 60 min).

Although different methods can be used to prescribe exercise intensity (42, 43), heart rate (HR) reserve was used in the current study to account for physiological adjustments that occur during prolonged aerobic exercise (e.g., cardiovascular drift) (44). The recommended quality (exercise intensity) and quantity (exercise volume) of aerobic exercise for participants were attained and exceeded gradually by increasing exercise intensity and duration (45). However, as a result of COVID-19 pandemic restrictions put in place, from the 4th week, the type of supervision was adapted to exclusively remotely supervised aerobic exercise (3 sessions per week). Weekly phone calls from the exercise specialist, who provided the weekly exercise prescription and tailored feedback based on the training logs, were used for supervision.

Both remotely and on-site supervised training sessions consisted of aerobic exercise (i.e., walking, running, or cycling). On-site aerobic exercise sessions (for the first 4 weeks) were performed using treadmill or stationary bikes. While the remotely supervised sessions were performed using treadmill or stationary bikes if the participant had them available at home or through other outdoor exercises (e.g., walking without the aid of tools) according to the possibilities and preferences of the participants. Regardless of the exercise modality, the sessions were performed at individualized exercise intensities (e.g., walking speed and grade or cycling wattage), allowing each participant to reach and maintain the prescribed target HR during the training sessions.

### 2.3 Sample collection and 16S rRNA gene (rDNA) sequencing

**Figure 1:**
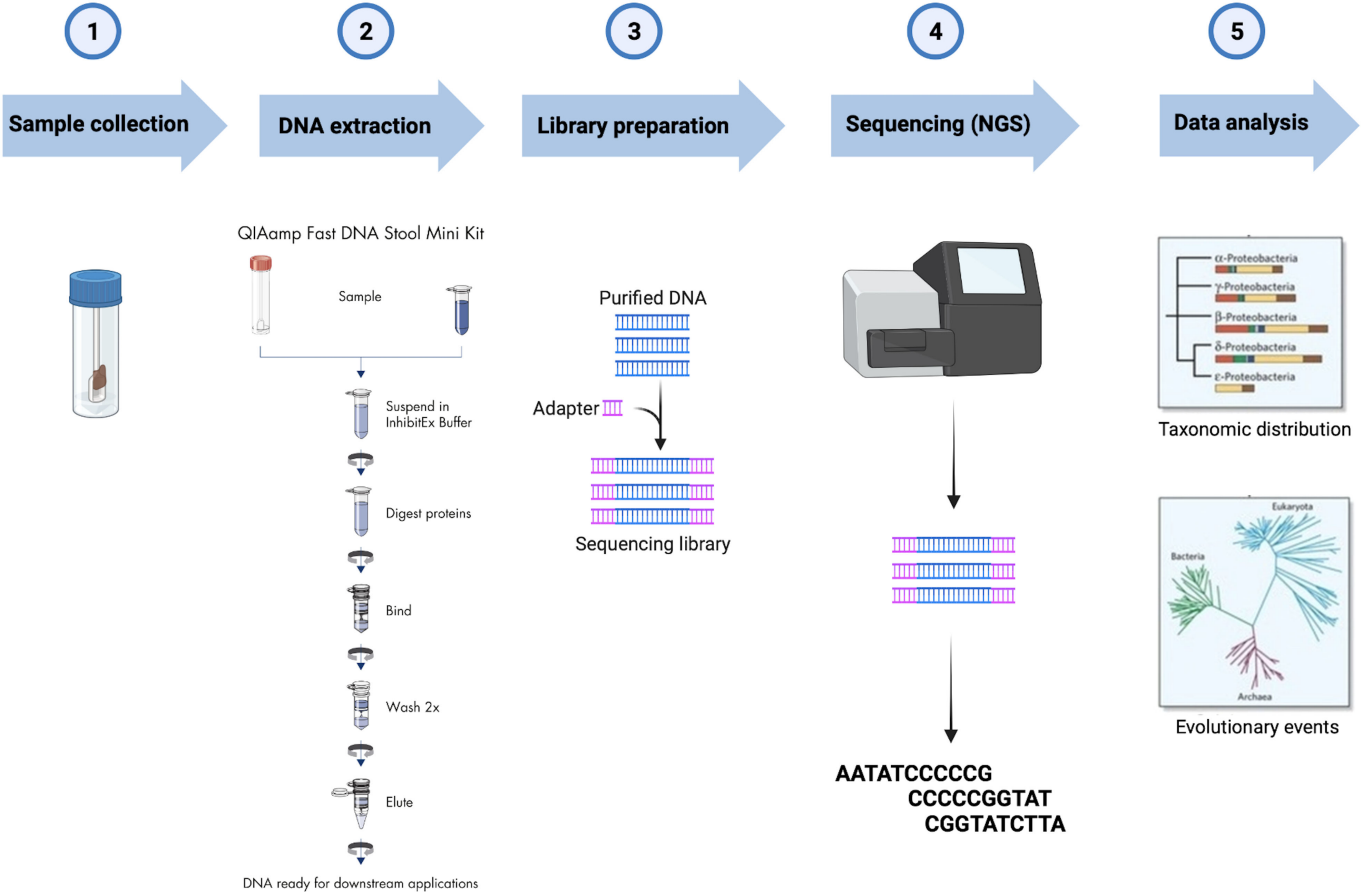
Workflow of the 16S rRNA gene analysis of fecal samples collected.

Fecal samples were collected at baseline and after 12 weeks in tubes filled with DNA stabilization buffer (CANVAX Biotech). For 16S rDNA sequencing, the total microbial DNA was extracted using the QIAamp PowerFecal DNA Kit (Qiagen) following the manufacturer’s protocol. After assessing DNA concentration and purity, samples were stored at -80° until processing. Library Preparation for the Illumina 16S Metagenomic Sequencing protocol was implemented starting from 12.5 ng of each DNA extract which was subjected to V3–V4 hypervariable regions of the bacterial 16S rDNA amplification using the universal primers previously published by Klindworth et al. (46) with Illumina adapters. Agencourt AMPure XP beads (Beckman Coulter, Milan, Italy) were utilized for the first PCR amplicons purification before the second round of PCR aimed to barcode the libraries using the Illumina dual-index system (Nextera XT Index Kit, Illumina Inc., San Diego, CA, USA) necessary for multiplexing. After the second purification step, the Qubit dsDNA BR Kit assay was employed to quantify the eluted DNA, which was then diluted to 4nM and pooled. Sequence data generated as FASTQ files, deposited in the Arrayexpress repository under accession code E-MTAB-12486. FASTQ raw sequencing data were imported into QIIME2 v.2021.244, and Illumina primers were removed using the q2-cutadapt plugin in trim-paired mode (47). Denoising of trimmed sequences was performed in paired-end mode using the q2-dada2 plugin (48). The q2-feature-classifier plugin was used to perform the taxonomy assignment of the amplicon sequence variants (ASVs) (49) based on the pre-trained Naïve Bayes classifier SILVA 138 99% operational taxonomic units (OTUs) full-length sequence dataset (50). The workflow from sample collection to data analysis is reported in **Figure 1**.

### 2.4 Statistical analyses

Data are reported as median and quartiles (Q1-Q3). The microbial alpha diversity indexes (OTUs number and Shannon’s Effective Number of Species (ENS)), which describe the richness of the microbial community (51), were calculated with the *diversity* function of the *vegan* R package, in pre- and post-intervention, and compared by a Wilcoxon signed-rank test for paired data. The choice for the use of Shannon’s effective number is that it describes the taxa to be considered important in a sample, and it is calculated by the exponential of the Shannon-Wiener index, which considers the differences in the abundance of each species. For further analysis, data were filtered for a relative abundance of 0.1% in both pre- and post-training conditions. A permutational analysis of variance (PERMANOVA) for repeated measures on a Bray-Curtis distance matrix was applied to test pre-post differences in the relative abundances at different taxonomic levels; *post-hoc* comparisons were conducted with a Wilcoxon test, and a false discovery rate (FDR) with Benjamini-Hochberg correction was applied to account for multiple comparisons. Effect sizes (r) were then calculated with the *rstatix* and *coin* R packages. To assess pre-post changes in global genera abundances, a non-metric dimensional scaling (nMDS) with Bray-Curtis distance was used. A multivariate analysis of variance for repeated measures (RM-MANOVA) was applied to test pre-post changes of physical and hematological variables. Lastly, a correlation plot was built to explore correlations between pre-post variations of genera abundances and variations of physical and hematological parameters; for graphical reasons, only significant correlations were reported (r_0.05,20_ ≥ 0.378). Statistical analyses were conducted using R Studio 4.1; the alpha value was set at the standard level of 0.05.

## 3 Results

### 3.1 Sample characteristics

The analysis included twenty women who were originally enrolled in the main trial (S1 Appendix). Baseline sample characteristics are described in **Table 1**. The mean age of the sample was 51.8 ± 7.8 years old, and the time since diagnosis was 10.2 ± 3.1 months.

**Table 1:** Clinical baseline characteristics of the sample (n = 20), reported as absolute and relative frequencies.

	n	%
Disease stage		
0	4	20%
I	10	50%
II	6	30%
III	–	–
Menopausal Status at diagnosis		
Pre-menopausal	3	15%
Post-menopausal	17	85%
Surgery type		
Mastectomy	3	15%
Quadrantectomy	16	80%
Lumpectomy	1	5%
Treatment in Addition to Surgery		
Radiation	7	35%
Chemotherapy	2	10%
Radiation + Chemotherapy	7	35%
None	4	20%
Current Endocrine Therapy		
Tamoxifen	5	25%
Aromatase inhibitor	10	50%
None	5	25%

Variations in cardiorespiratory fitness, body composition, blood biomarkers, and MD adherence are reported in **Table 2**. Significant changes in the post-intervention measures were detected for maximal oxygen uptake (*V̇*O_2max_), fasting glucose, testosterone concentration, HOMA, triglycerides, LDL, and adherence to MD [MeDiet Score by DianaWeb, as reported in Natalucci et al. (41)].

**Table 2:** Variations in cardiorespiratory fitness, body composition, and blood biomarkers.

	Pre	Post	*p*(*F*) ( $\eta_{\text{p}}^{2}$ )
**BMI (kg/m^2^)**	23.99 ± 2.74	23.72 ± 2.32	0.311 (0.054)
**Fat mass (%)**	29.15 ± 4.78	29.11 ± 3.76	0.952 (0.000)
***V̇*O_2max_ (ml·kg^-1^·min^-1^)**	32.55 ± 4.54	35.78 ± 6.07	<0.001 (0.617)
**Fasting glucose (mg/dL)**	100.15 ± 10.84	90.45 ± 11.55	<0.001 (0.597)
**Testosterone (ng/mL)**	0.28 ± 0.14	0.21 ± 0.17	0.018 (0.259)
**Insulin (microU/mL)**	6.99 ± 4.27	5.69 ± 3.73	0.091 (0.143)
**HOMA index**	1.81 ± 1.44	1.32 ± 1.02	0.036 (0.211)
**Progesterone (ng/mL)**	0.47 ± 0.25	0.47 ± 0.22	0.977 (0.000)
**Triglycerides (mg/dL)**	102.60 ± 43.68	87.02 ± 43.29	0.018 (0.263)
**Total cholesterol (mg/dL)**	207.10 ± 31.99	197.85 ± 33.31	0.119 (0.123)
**HDL (mg/dL)**	59.15 ± 14.37	58.10 ± 11.89	0.539 (0.020)
**LDL (mg/dL)**	129.90 ± 23.44	120.75 ± 26.98	0.022 (0.246)
**hs-CRP (mg/L)**	1.30 ± 0.84	1.07 ± 0.88	0.078 (0.155)
**Mediterranean diet score**	7.65 ± 2.32	9.25 ± 2.38	0.002 (0.406)

BMI, body mass index; V̇O_2max_, maximal oxygen uptake; HDL, high-density lipoprotein; LDL, low-density lipoprotein; hs-CRP, high-sensitivity C-reactive protein; Mediterranean diet score: MeDiet Score by DianaWeb.

### 3.2 Microbial composition

**Figure 2:**
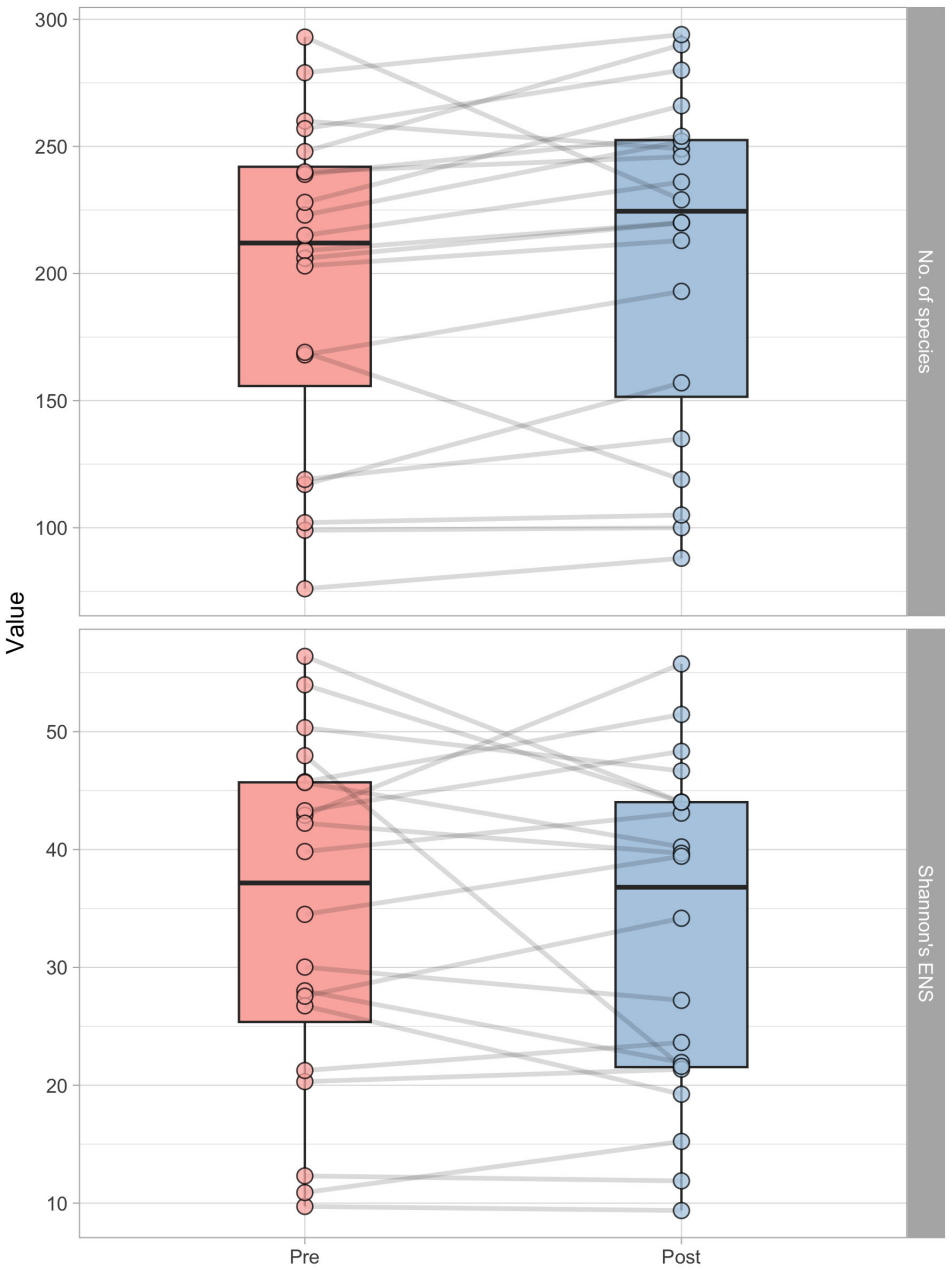
Boxplots representing the number of operational taxonomic units (OUT’s number, above) and Shannon’s effective number of species (ENS, below) in pre- and post-intervention, respectively. Each point represent a subject, and grey lines connect pre and post-intervention measurements of each participant.

After the lifestyle intervention, as shown in **Figure 2**, there was a significant (p=0.025) increase in OTUs number. The pre-intervention count was 212.0 (Q1: 155.8; Q3 = 242.0), while the post-intervention number was 224.5 (Q1: 151.5; Q3: 252.2), which represents a 5.7% increase. However, there was no significant (p=0.620) change in the effective number of species (ENS) as measured by Shannon’s ENS index. The ENS count remained relatively constant, with a pre-intervention count of 37.2 (Q1: 25.4; Q3: 45.7) and a post-intervention count of 36.8 (Q1: 21.5; Q3: 44.2).

**Figure 3:**
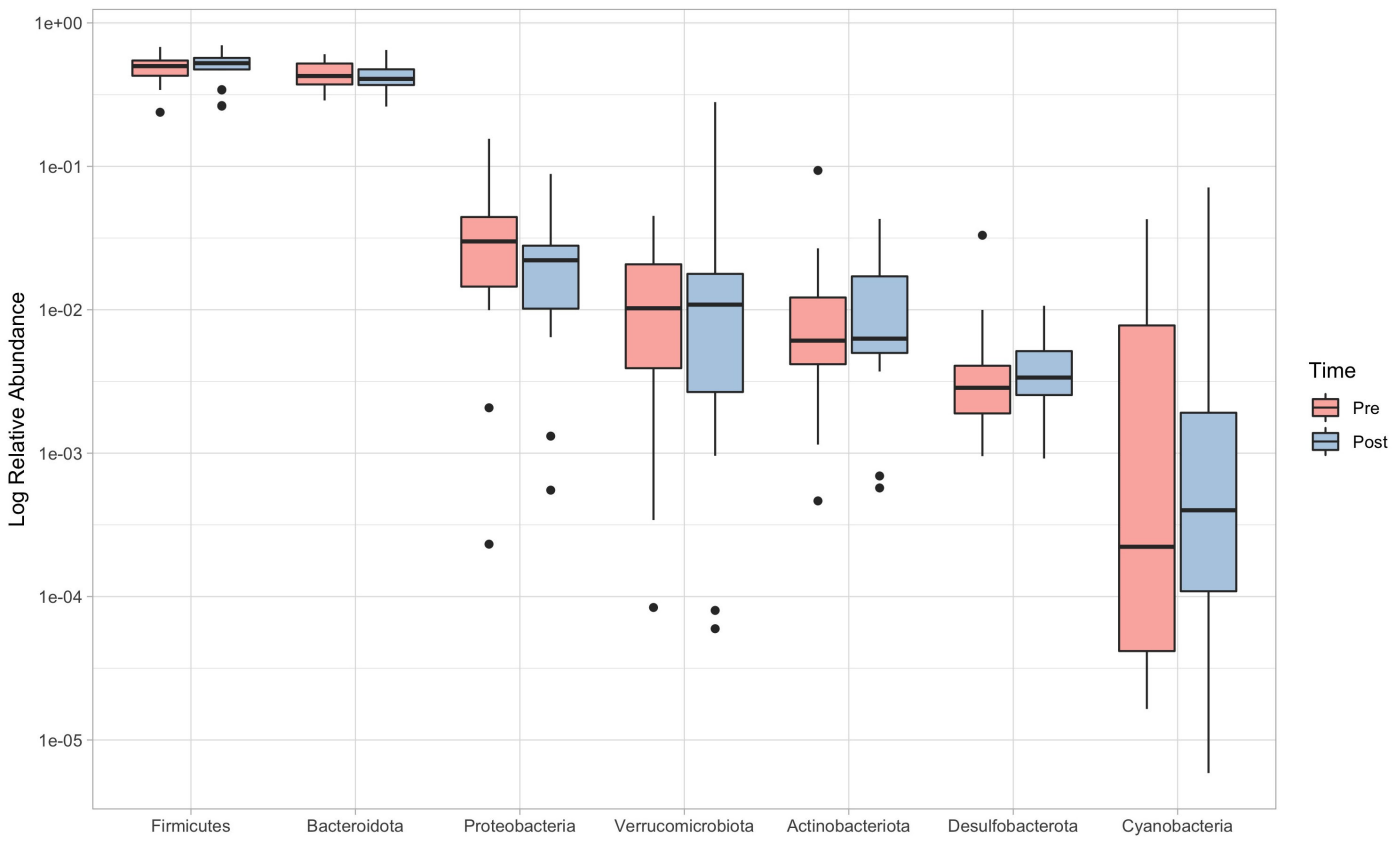
Relative abundances (log scale) of the seven more representative phyla, in order of abundance. Boxes limits represent 25th and 75th percentiles, and the black line inside the box represents the median. Single dots represent outliers individual data, which fall outside the 95% confidence interval.

PERMANOVA revealed significant differences between pre and post-intervention at phylum (F=0.62; p=0.035) and order levels (F=0.51; p=0.036). *Post-hoc* comparisons at the phylum level detected a significant reduction in the abundance of Proteobacteria (pre: 3.01% (1.45-4.43), post: 2.22% (1.03-2.80); V=189; p=0.006, ES=0.701), while no significant changes were reported for any of the other phyla (**Figure 3**).

Although not significant (p=0.067), a moderate effect-size (ES=0.410) increase in the Firmicutes/Bacteroidetes (F/B) ratio could be noticed, which showed values of 1.19 (0.82-1.43) in the pre-intervention and 1.24 (0.99-1.55) in the post-intervention.

At the order level, significant changes were observed for Lactobacillales (p=0.040, ES=0.459), Acidaminococcales (p=0.048, ES=0.442), and Burkholderiales (p=0.005, ES=0.609). However, these p-values become not significant when applying FDR correction for multiple comparisons (p=0.380 for Lactobacillales and Acidaminococcales, p=0.110 for Burkholderiales).

**Figure 4:**
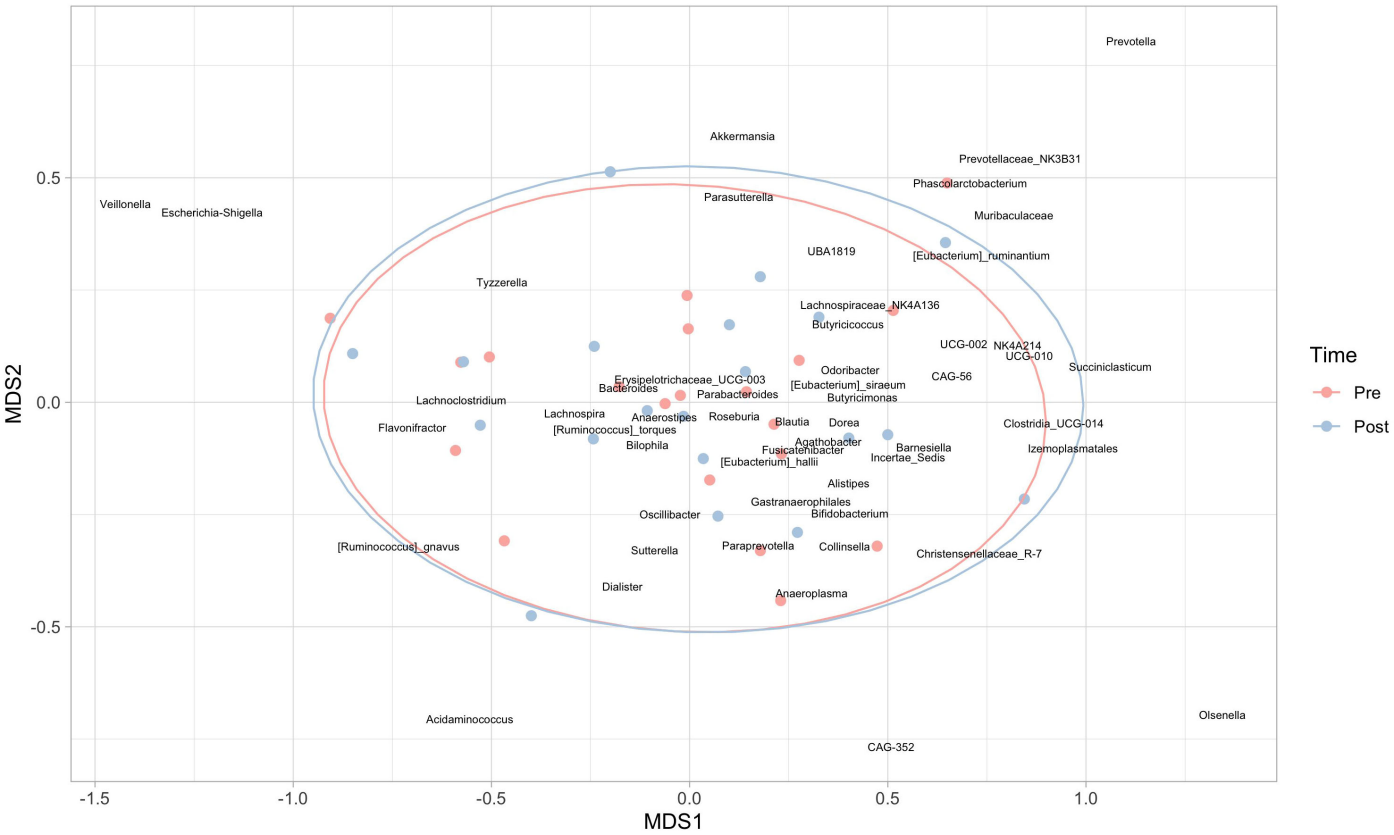
Non-metric multidimensional scaling (nMDS) at the genera level. Red points represent subjects in the pre-intervention, while blue dots refer to the post-intervention; 95% confidence ellipses are also represented.

At the genus level, significant decreases in *Odoribacter* (p=0.024), *Erysipelotrichaceae_UCG-003* (p=0.020), *Coprococcus* (p=0.042), *Lachnospiraceae_UCG-004* (p=0.015), *Sutterella* (p=0.007) and a significant increase in *Colidextribacter* (p=0.044) were observed. However, when an exploratory non-metric multidimensional scaling was used to compare global genera abundances at the two times, ellipses were almost perfectly overlapping (**Figure 4**), supporting the absence of significant results in the PERMANOVA.

### 3.3 Correlations with physical variables

**Figure 5:**
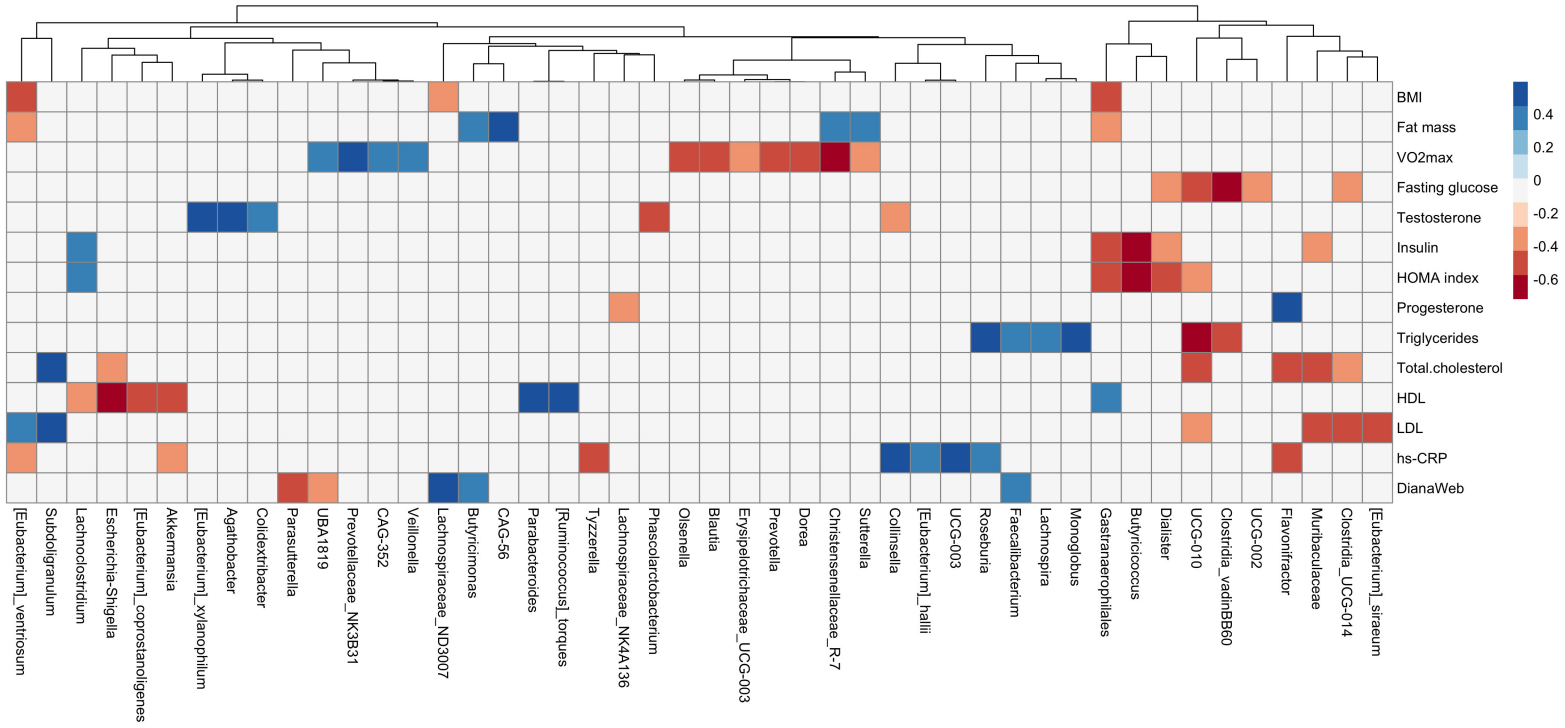
Correlation plot between delta (post-pre) values of anthropometric and cardiometabolic variables (body composition, cardiorespiratory fitness, and blood biomarkers) and genera relative abundances. Column clustering among genera is reported. Blue color indicates positive correlation, red color negative correlation. Only variables showing significant correlations are reported (Spearman r_0.05,20_ ≥ 0.378).

A correlation matrix has been used to explore associations between variations of anthropometric and cardiometabolic variables and genera abundances (**Figure 5**). Negative correlations were reported between fasting glucose and *Clostridia_vadinBB60* (r=-0.62), insulin and HOMA index and *Butyricicoccus* genera (r = -0.72 and -0.66, respectively), and HDL cholesterol and *Escherichia/Shigella* (r = -0.59). Moreover, positive correlations were found between MD adherence and *Lachnospiraceae_ND3007* (r = 0.50), *Faecalibacterium* (r = 0.38), and *Butyricimonas* (r = 0.39).

## 4 Discussion

The risk of cancer recurrence and the increased risk of chronic diseases brought on by cancer treatments endanger the well-being of cancer survivors (52). Improving lifestyle behaviors attenuates these risks, and in particular, nutrition and physical activity have been considered powerful allies to cancer patients and survivors, as they positively impact body mass, physical fitness, fatigue, depressive symptoms, anxiety, inflammatory profile, and quality of life (53–57). Inversely, unhealthy eating patterns and a sedentary lifestyle are the main contributors to metabolic syndrome, which is associated with chronic low-grade systemic inflammation and symptoms in patients with BC who have undergone chemotherapy and survivors (58–60).

Diet and exercise are known to alter the composition of the gut microbiota, with resulting health-improving effects (61, 62). Up to date, a limited number of human studies have uncovered that exercise could play a beneficial role in gut health, increasing microbial diversity and changing microbiota composition depending on training intensity and duration (29). Only a few clinical studies investigated the effects of dietary interventions on the gut microbiome and/or metabolome in patients with cancer (63).

In this study, we investigated the impact of a home-based lifestyle intervention on the gut microbiota of BC survivors, demonstrating remarkable changes. In particular, a significant decrease in Proteobacteria has been observed. This phylum is normally associated with the instability of the microbial community that can lead to dysbiosis or disease (64). Furthermore, *Escherichia/Shigella* bacteria belonging to the Proteobacteria phylum possess *β*-glucuronidase enzymes, which produce free estrogens from conjugated estrogens derived from the liver; free estrogens can re-enter the bloodstream and reach different organs, including the breast (65).

Physical exercise has been previously reported to reduce inflammatory processes, decreasing Proteobacteria but also increasing Actinobacteria (29). Though representing only a small percentage of the total microbial community, Actinobacteria play a key role in maintaining the homeostasis of the gastrointestinal tract by increasing the expression of occluding junctions, regulating the synthesis and catabolism of mucins, providing energy for epithelial cell proliferation, and stimulating the immune system. In particular, Bifidobacteria (belonging to the phylum Actinobacteria) produce lactate, which can be used by a group of microorganisms defined as “lactate users” to produce butyrate, an SCFA that plays an important role in regulating energy metabolism and insulin sensitivity (66). Moreover, the F/B ratio increased after the lifestyle intervention, although not statistically significant. This result agrees with other studies in which increases in the F/B ratio occur following physical activity (24, 29) and is particularly important since Firmicutes are the main producers of SCFA butyrate. Furthermore, a relationship between cardiorespiratory fitness (*V̇*O_2max_) and F/B ratio in healthy young adults was also reported by Durk et al. (67). A slight but not significant increase was observed in Actinobacteria and Bifidobacteria after 12 weeks of a home-based lifestyle intervention in BC survivors, which exerts an inflammatory and beneficial metabolic effect (68). Interestingly, at the order level, Lactobacillales were found to be significantly increased after the lifestyle intervention. This order includes bacteria belonging to the genus of *Lactobacillus*, which are microorganisms with anti-inflammatory functions and may have a role against BC, as already suggested (69). Moreover, the lifestyle intervention produced a decrease in *Sutterella*, a bacterial genus previously found enriched in patients with BC (70). The correlation analysis between bacterial genera and anthropometric and cardiometabolic variables evidenced a strong positive association between adherence to MD and the butyrate producers *Lachnospiraceae_ND3007*, *Faecalibacterium*, and *Butyricimonas*, in agreement with previous studies (71, 72).

An increase in the consumption of dried fruit and cereal-based meals, and a reduction of red meat, desserts, and glasses of wine per week (data not shown) have been observed in all participants. The improvement of the MeDiet score obtained from the lifestyle intervention is particularly interesting for glycemic control, as highlighted by the amelioration of metabolic and inflammatory parameters (**Table 2**). The MD has been linked to improved health, including the production of SCFAs and anti-inflammatory properties that reduce the risk of chronic inflammatory diseases such as type II diabetes (73–75).

Another butyrate-producing microorganism, *Butyricicoccus*, was negatively correlated with insulin levels and the HOMA index. This result is in line with previous studies demonstrating that butyrate acts as an insulin-sensitizing agent (76, 77). Moreover, *Butyricicoccus* was previously found to be enriched in physically active people with respect to less active individuals, as reported by Magzdal et al. (78). The same was observed for *Clostridia_vadinBB60* which, in our study, was negatively correlated with fasting glucose. An interesting inverse association was found between the pro-inflammatory bacteria *Escherichia/Shigella* and HDL cholesterol.

The present study shows that a lifestyle intervention based on physical activity and adherence to the MD in patients with BC was able to shape gut microbiota towards a healthier profile by modulating some microorganisms capable of decreasing inflammation and others capable of improving the lipid and glycemic assets of the host. The study recognizes certain limitations. Due to the relatively small sample size, the data are preliminary and should be interpreted with caution. Also, the absence of a control group should be acknowledged. Even though significant changes have been noted, it is still unknown whether also tiny modifications have any biological significance. Nonetheless, it’s among the earliest studies which demonstrate how a lifestyle intervention in previously sedentary BC survivors can lead to an improvement in the composition of their gut microbial community. In order to gain a more comprehensive understanding of this topic, further research is required.

This study is part of the MoviS Trial (NCT04818359), a randomized controlled trial on the effect of aerobic exercise training on quality of life among BC survivors starting in January 2020 and ending in 2025. The forced changes in the study protocol, imposed by COVID-19 pandemic restrictions, made the difference between IA and CA interventions for the first recruited group of BC survivors negligible, and the results of the two groups were combined [Natalucci et al., 2021 (41)] qualifying this study as a pre-post assessment. Although the lack of a control group represents a limitation, no healthy control group, and no No-Covid control group were included, further studies on the MoviS cohort will allow a comparison of these preliminary data obtained on gut microbiota and verify whether some other factors (e.g., Covid-19 restrictions) could better explain the changes in the gut microbiota induced by a 12-weeks lifestyle intervention in this particular BC survivors population.

In this study, we enrolled BC survivors at high risk of recurrence due to their sedentary condition, metabolic syndrome, inflammatory level, and dysmetabolism in the pandemic emergency, and we verified the effect of a lifestyle intervention in the improvement of cardio-metabolic condition and microbiota in a pre- and post-intervention assessment.

The elaborations presented in this study should be contextualized, taking into account the type of BC survivor population in Italy during the first Covid lockdown that tended to aggravate the BC health status by worsening their lifestyle and, consequently, their prognosis. In this line, we verified, for example, the profile of sedentary behavior among 781 BC survivors belonging to the cohort of the Italian DianaWeb project in the period of the first Covid lockdown. We observed a trend toward a decrease in physical activity (22%, 57%, and 26% for walking activity, vigorous activity, and total PA, respectively), and the sitting and lying time increased up to 54.2% [Natalucci et al., 2021 (40)], suggesting how COVID-19 emergency increased the unhealthy behaviors in BC patients, indicative of a possible higher risk of worse prognosis.

Our findings suggest that adopting a healthy lifestyle, by affecting the gut microbiota, may help to improve some biological parameters, such as the reduction of the inflammatory pattern involved in health maintenance, improvement of the prognosis, and reduction of the recurrence risk.

## Data availability statement

The datasets presented in this study can be found in online repositories. The names of the repository/repositories and accession number(s) can be found below: https://www.ebi.ac.uk/arrayexpress/, E-MTAB-12486.

## Author contributions

Conceptualization: SDZ, RE and EBa. Sample collection: VN, SDZ, DA and LV. Metagenomic experiments and analyses: VP, FP, AV, EBi, CP, GM, LC and EV. Clinical parameters evaluation: VN, CFM, FL, RE and VC. Statistical analyses: SA, DS and MR. Supervision: GP, VS, RE and EBa. Writing – Original draft: SDZ, VN, DA, SA, GA, VP, FP, LC and EBa. Writing – Review and Editing: All authors. All authors contributed to the article and approved the submitted version.
