# Supplementary figures and images for: Changes in gut microbiota composition after 12 weeks of a home-based lifestyle intervention in breast cancer survivors during the COVID-19 lockdown
Source: Front Oncol. 2023 Sep 1;13:1225645. doi: 10.3389/fonc.2023.1225645 (PMC10505708; doi:10.3389/fonc.2023.1225645)

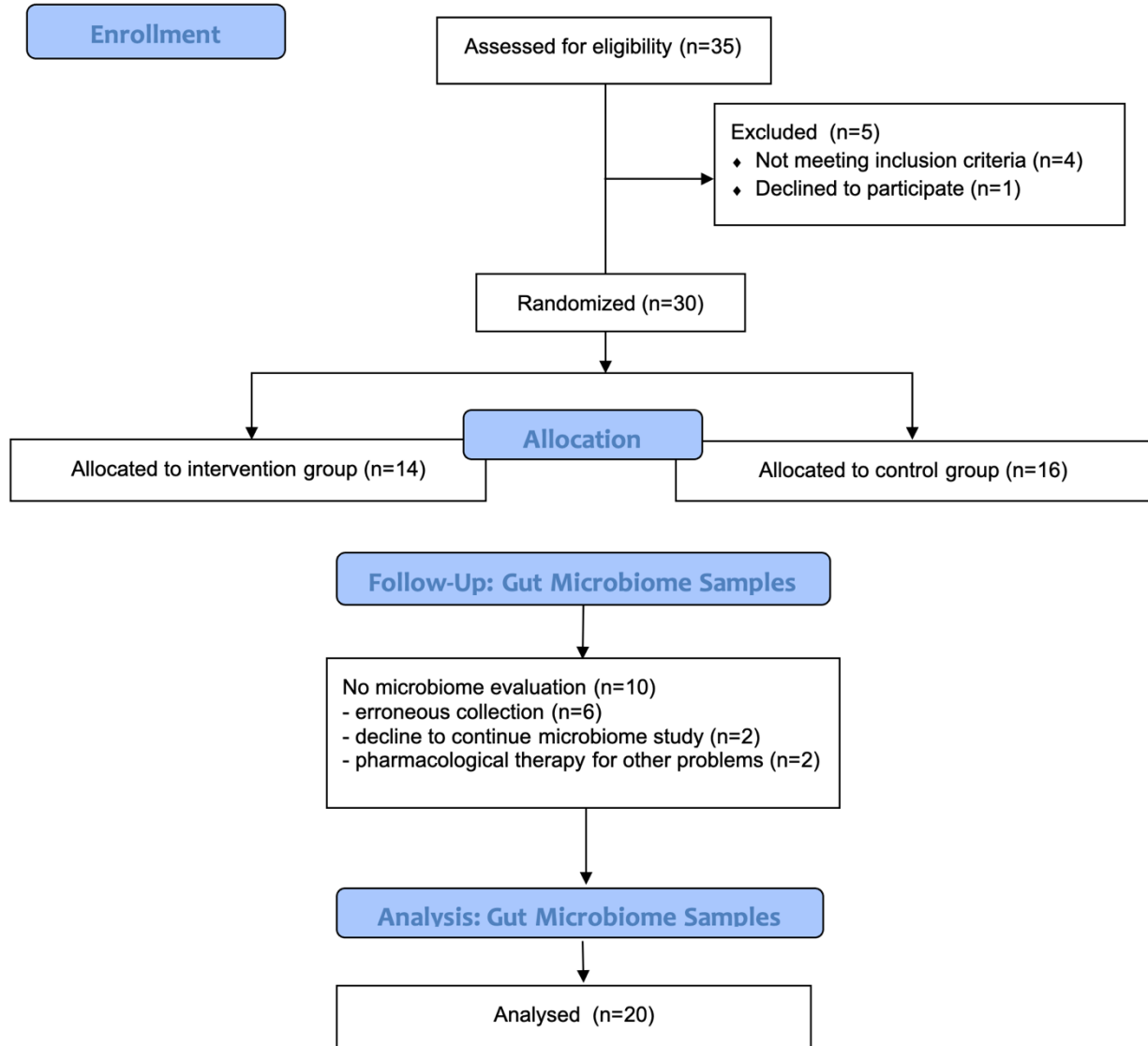

**Supplementary Figure 1.** CONSORT 2010 Flow Diagram.

Supplement: Supplementary file 1 [file Image_1.pdf]
